# The Evaluation of Two Colistin Minimum Inhibitory Concentration (MIC) Methods: A Comparative Study of MicroScan Walkaway 96 Plus ID/AST and Colistin Broth Disc Elution Test at a Tertiary Care Hospital in Punjab

**DOI:** 10.7759/cureus.99786

**Published:** 2025-12-21

**Authors:** Niya Roy, Shereen R Varghese, Sangeetha Mohan, Mani Bhushan Kumar, Gulfishan

**Affiliations:** 1 Microbiology, Christian Medical College and Hospital, Ludhiana, IND

**Keywords:** colistin, colistin broth disc elution, major errors, microscan, very major errors

## Abstract

Background and objective

The rise of multidrug-resistant (MDR) Gram-negative bacteria poses significant challenges in clinical settings, especially concerning colistin, a last-resort antibiotic. This study sought to compare two colistin minimum inhibitory concentration (MIC) testing methods: the automated MicroScan Walkaway 96 Plus AST system and the colistin broth disc elution (CBDE) test, particularly in (MBL)-producing Gram-negative organisms. The study aimed to evaluate the incidence of major errors (MEs) and very major errors (VMEs) in colistin susceptibility results between the two methods and to assess the comparative reliability and accuracy of these techniques for detecting colistin resistance.

Materials and methods

A study was conducted from October 2022 to September 2023 at a tertiary care hospital in Ludhiana, Punjab, involving 105 Gram-negative bacterial isolates from patient samples, including pus, blood, body fluids, and urine. Colistin susceptibility was assessed using the MicroScan Walkaway 96 Plus system and the CBDE method. Errors in susceptibility results were categorized as MEs and VMEs. Descriptive statistics were used for categorical and quantitative variables. Kappa agreement was calculated to evaluate the agreement between the two methods. Statistical analysis was performed using IBM SPSS Statistics software (IBM Corp., Armonk, NY), with a significance level set at p < 0.05.

Results

The study found that 86.67% of isolates showed intermediate susceptibility by MicroScan, compared to 95.24% using the CBDE test. A total of 10 MEs (9.52%) and one VME (0.95%) were identified. The study also observed discrepancies in colistin resistance rates, particularly for *Acinetobacter baumannii (A. baumannii)* and *Klebsiella pneumoniae (K. pneumoniae).*

Conclusions

MicroScan demonstrated a moderate agreement with the CBDE test in colistin susceptibility testing, with notable MEs and VMEs. These discrepancies highlight the need for further validation and the complementary use of both methods to ensure accurate susceptibility testing and effective clinical management of MDR infections.

## Introduction

Gram-negative bacteria pose a formidable challenge in clinical settings, frequently emerging as important nosocomial pathogens with a strong tendency to develop resistance to essential antibiotics such as carbapenems, monobactams, aminoglycosides, and fluoroquinolones [1]. The global rise in colistin resistance has heightened concerns about infections caused by Gram-negative organisms that may become virtually untreatable [2].

In this study, we focused on Gram-negative organisms producing metallo-beta-lactamases (MBLs). MBLs represent a significant therapeutic challenge due to their capacity to hydrolyse a broad range of beta-lactam antibiotics, including carbapenems, thereby rendering these drugs ineffective. Their enzymatic activity contributes to the emergence of multidrug-resistant (MDR) strains, severely limiting treatment options and complicating patient management. Despite their clinical importance, standard susceptibility testing methods may fail to detect MBL production, especially in isolates that appear carbapenem-susceptible. This creates a diagnostic dilemma, as the accurate identification of MBL-producing Gram-negative bacteria is crucial for guiding appropriate therapy and preventing treatment failure [2].

Gram-negative isolates identified as MBL producers through phenotypic methods subsequently underwent in vitro colistin susceptibility testing using the colistin broth disc elution (CBDE) test [3]. Their results were then compared with those obtained from the automated Microscan ID/AST system. The study aimed to evaluate the incidence of major errors (MEs) and very major errors (VMEs) to identify discrepancies in colistin susceptibility results. MEs were defined as isolates showing intermediate susceptibility by CBDE (false-resistant result) and resistant results using the Microscan ID/AST system. VMEs were defined as isolates resistant by CBDE but intermediate by Microscan (false-intermediate result) [4].

Accurate colistin susceptibility testing is vital for several reasons. First, it enables clinicians to choose the most effective antibiotic therapy for infections caused by MDR Gram-negative bacteria, especially when colistin is the last available treatment option. Second, precise testing helps prevent treatment failures and the progression of severe infections when therapeutic choices are limited. Third, it plays a key role in antimicrobial stewardship by preventing unnecessary or inappropriate colistin use, thus preserving its effectiveness and reducing the emergence of resistance. Additionally, accurate results enhance patient safety by avoiding adverse effects such as colistin-induced nephrotoxicity due to incorrect susceptibility interpretations. Finally, reliable susceptibility data support global surveillance initiatives tracking antimicrobial resistance patterns, facilitating research into resistance mechanisms and the development of novel therapeutic agents [5-7].

Specifically, this study aimed to generate reliable colistin susceptibility data by comparing the performance of the Microscan ID/AST system with the CBDE test in MBL-producing Gram-negative isolates. We believe the findings will offer valuable guidance to clinicians in determining appropriate colistin dosing for critically ill patients requiring targeted therapy.

## Materials and methods

A prospective study involving a minimum of 100 patient samples was conducted from October 2022 to September 2023 in the Department of Microbiology at a tertiary care hospital in Ludhiana, Punjab. The study included 105 Gram-negative bacterial isolates identified as MBL producers through phenotypic detection. These isolates were obtained from various clinical samples, including 69 from pus and body fluids, 29 from urine, and seven from blood. Phenotypic detection of MBL production was performed using the combined disc test (CDT) and the modified Hodge test. Isolates that could not be phenotypically confirmed were excluded from further analysis.

**Figure 1:**
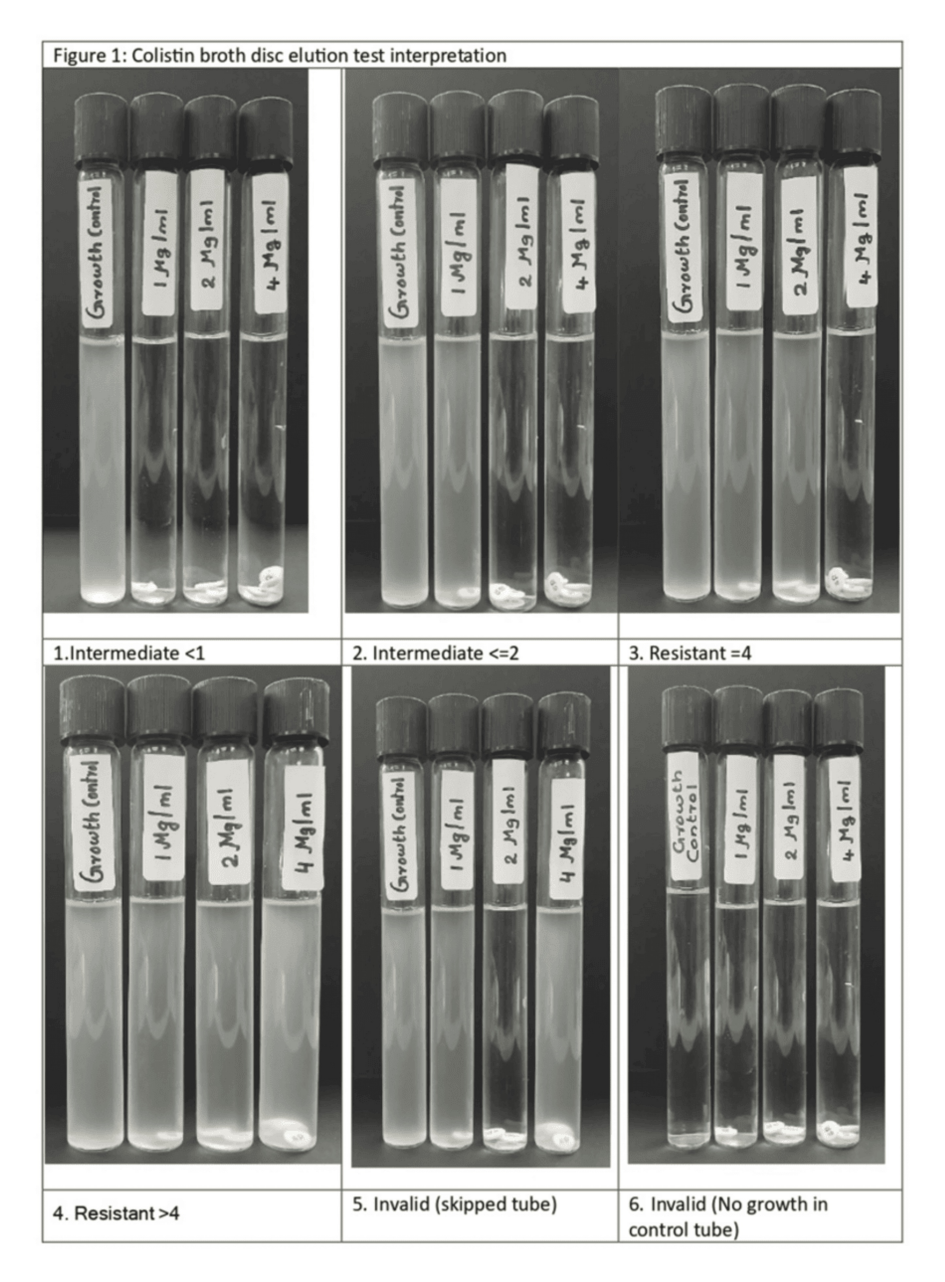
Colistin broth disc elution test interpretation

In vitro colistin susceptibility was assessed using the Microscan Walkaway 96 Plus AST system and the CBDE test. Antibiotic susceptibility results, including those for colistin, were interpreted according to Clinical and Laboratory Standards Institute (CLSI) guidelines, categorizing bacterial isolates as intermediate (I) or resistant (R) [3,5]. *Pseudomonas aeruginosa (P. aeruginosa)* (ATCC 273583) and *Escherichia coli (E. coli)* (ATCC 25922) were used as quality control strains [8]. Figure 1 illustrates the colistin broth disc elution test interpretation.

Discrepancies in colistin susceptibility results were classified into two categories: VMEs and MEs. VMEs occurred when bacterial isolates were reported as intermediate by the MicroScan WalkAway 96 Plus system but as resistant by the CBDE method, which is a critical concern because it may result in the inappropriate use of colistin despite actual resistance. Conversely, MEs were identified when isolates were classified as resistant by MicroScan but as intermediate by CBDE, potentially limiting the use of colistin even when it could still be clinically effective, thereby affecting treatment decisions.

Statistical analysis

Categorical variables were presented as counts and percentages (%), whereas quantitative data were expressed as mean ± standard deviation (SD) and as median with interquartile range (IQR) (25th-75th percentiles). Inter-rater kappa statistics were used to assess the level of agreement between the Colistin MicroScan WalkAway 96 Plus AST system (minimum inhibitory concentration (MIC) and the CBDE (MIC) test. Data entry was performed using Microsoft Excel, and the final analysis was conducted using IBM SPSS Statistics software, version 25.0 (IBM Corp., Armonk, NY). A p-value <0.05 was considered statistically significant.

## Results

Age and gender

Out of 105 patients, 61 (58.10%) were aged over 40 years, while 29 (27.62%) fell into the age group of 20-40 years. Only 15 patients (14.29%) were younger than 20 years. The mean age of the study subjects was 44.41 years, with a median (25th-75th percentile) of 48 (33-58); 64 (60.95%) were males, while 41 (39.05%) were females.

Organism-wise distribution

A diverse range of organisms was isolated from the 105 samples. The distribution was as follows: *Acinetobacter baumannii (A. baumannii)* complex in 32 (30.48%) cases, *Klebsiella pneumoniae (K. pneumoniae)* subsp. *pneumoniae* in 27 (25.71%) cases, *E. coli *in 22 (20.95%) cases, *P. aeruginosa* in 13 (12.38%) cases, *Enterobacter cloacae (E. cloacae)* in eight (7.62%) cases, *Citrobacter freundii (C. freundii)* in two (1.90%) cases, and *K. pneumoniae* subsp. *rhinoscleromatis* in one (0.95%) case (Table 1). The most commonly isolated organism was the *A. baumannii* complex.

**Table 1:** Organism-wise distribution of all 105 Gram-negative bacterial isolates

Organisms	Frequency	Percentage
*Acinetobacter baumannii* complex	32	30.48%
*Klebsiella pneumoniae* subsp. *pneumoniae*	27	25.71%
Escherichia coli	22	20.95%
Pseudomonas aeruginosa	13	12.38%
Enterobacter cloacae	8	7.62%
Citrobacter freundii	2	1.90%
*Klebsiella pneumoniae* subsp. *rhinoscleromatis*	1	0.95%
Total	105	100.00%

Colistin resistance

The results of colistin susceptibility testing using the MicroScan WalkAway 96 Plus AST system and the CBDE method were analyzed across different bacterial species. Among the 32 cases of *A. baumannii* complex, six (18.75%) were reported as resistant by the MicroScan method, whereas two (6.25%) were resistant according to CBDE. No VMEs were observed. However, four (12.50%) cases showed MEs, resulting in moderate agreement (kappa = 0.448, p = 0.002) and a categorical agreement of 87.5%.

Among the 27 cases of *K. pneumoniae *subsp. *pneumoniae*, five (18.52%) were identified as resistant by the MicroScan method, while one (3.70%) was deemed resistant by CBDE, producing four (14.81%) MEs. The kappa value was 0.289 (p = 0.033), indicating fair agreement, with a categorical agreement of 85.19%. In the case of *E. coli*, a discrepancy was noted: one (4.55%) isolate was resistant by CBDE, while none were resistant by the MicroScan method. This resulted in one (4.55%) VME. Among the eight cases of *E. cloacae*, three (37.5%) were resistant by the MicroScan method and one (12.5%) by CBDE, resulting in two (25%) MEs. The kappa coefficient was 0.385 (p = 0.168), indicating moderate agreement, with a categorical agreement of 75%. For *C. freundii*, both isolates were fully susceptible per both methods, and no errors were recorded. Similarly, no errors or resistance were observed in cases of *K. pneumoniae* subsp. *rhinoscleromatis* and *P. aeruginosa *(Table 2).

**Table 2:** Resistance pattern of colistin MicroScan Walkaway 96 plus AST system (MIC) and CBDE (MIC) test MIC: minimum inhibitory concentration; CBDE: colistin broth disc elution; VME: very major error; ME: major error

Organism	MicroScan (MIC), n (%)	CBDE (MIC), n (%)	VME, n (%)	ME, n (%)	κ (Kappa) (p-value)	Categorical agreement (%)
*Acinetobacter baumannii* complex (n = 32)	6 (18.75%)	2 (6.25%)	0 (0%)	4 (12.50%)	0.448 (0.002)	87.50%
*Klebsiella pneumoniae* subsp. *pneumoniae* (n = 27)	5 (18.52%)	1 (3.7%)	0 (0%)	4 (14.81%)	0.289 (0.033)	85.19%
*Escherichia coli* (n = 22)	0 (0%)	1 (4.55%)	1 (4.55%)	0 (0%)	-	95.45%
*Pseudomonas aeruginosa* (n = 13)	0 (0%)	0 (0%)	0 (0%)	0 (0%)	-	100%
*Enterobacter cloacae* (n = 8)	3 (37.5%)	1 (12.5%)	0 (0%)	2 (25%)	0.385 (0.168)	75%
*Citrobacter freundii* (n = 2)	0 (0%)	0 (0%)	0 (0%)	0 (0%)	-	100%
*Klebsiella pneumoniae* subsp. *rhinoscleromatis* (n = 1)	0 (0%)	0 (0%)	0 (0%)	0 (0%)	-	100%

Errors

Regarding errors in testing, 94 out of 105 cases showed no discrepancies. MEs were observed in 10 cases (9.52%), while VMEs were identified in only one case (0.95%). In total, one VME and 10 MEs were noted (Table 3). Among the 10 MEs, three patients received polymyxin B, and one patient received colistin. Regarding the single VME, the patient had not received colistin before sample collection; instead, he had been administered meropenem and teicoplanin. The patient, a 58-year-old male, had *E. coli *isolated from his urine and was diagnosed with sepsis. Based on multiple culture reports and clinical deterioration, he was subsequently initiated on colistin therapy.

**Table 3:** Categorization of discrepancies in colistin susceptibility: MEs vs. VMEs ME: major error; VME: very major error

Categorization	Frequency	Percentage
ME		
No	95	90.48%
Yes	10	9.52%
VME		
No	104	99.05%
Yes	1	0.95%

## Discussion

The current study was conducted in the Department of Microbiology over one year, from October 2022 to September 2023. It included 105 bacterial isolates that were phenotypically identified as MBL-producing Gram-negative bacteria from inpatients at our institution. Bacterial isolates were obtained from various samples received in the Microbiology Department, including pus and body fluids (such as wound swabs, high vaginal swabs, ascitic fluid, peritoneal fluid, perirenal fluid, and sputum), as well as blood and urine. Only isolates that tested positive for both the CDT and the modified Hodge test were included. Isolates that showed resistance to the imipenem-EDTA combination in the combined disc method, along with those demonstrating pan-resistance, were excluded from the study. The results of the study are presented below.

In the present study, the mean age of the subjects was 44.41 years. Among the participants, 64 (60.95%) were male, while 41 (39.05%) were female. In a study conducted by Vamsi et al. on MBL production in Gram-negative bacterial isolates, 131 (60.3%) of the 217 MBL-producing isolates were from male patients, while 86 (39.6%) were from female patients. The study also revealed that the highest proportion of isolates was from patients aged zero to nine years (54.3%), followed by those aged 40-49 years (13.3%) and 60 years (11.9%) [9].

According to research by Chakraborty et al. concerning infections in critical care unit patients caused by Gram-negative bacteria producing MBL, patients with infections due to MBL-positive organisms were predominantly aged between 61 and 80 years [10]. In another study by Panigrahi et al., the median age of patients with MDR Gram-negative bacterial isolates from different clinical samples in the ICU was 54.81 years, with a higher proportion of males [11]. Additionally, Singh et al. included 25 patients with a mean age of 45.09 ± 17.59 years in their study comparing the MicroScan with Mikrolatest broth microdilution (BMD) [1]. In the present study, the isolated organisms included *A. baumannii* complex in 32 cases (30.48%), *K. pneumoniae* subsp. *pneumoniae* in 27 cases (25.71%), *E. coli* in 22 cases (20.95%), *P. aeruginosa *in 13 cases (12.38%), *E. cloacae *in eight cases (7.62%), *C. freundii *in two cases (1.90%), and *K. pneumoniae* subsp. *rhinoscleromatis* in one case (0.95%). The most common organism isolated was *A. baumannii* complex.

In a study by Vamsi et al. investigating MBL production in Gram-negative bacterial isolates, among the 217 isolates that produced MBL, the distribution of MBL-producing Gram-negative bacteria was as follows: 114 (97.3%) were *K. pneumoniae*, 61 (95.3%) were *E. coli *isolates, 17 (73.9%) were *Acinetobacter* isolates, and 14 (73%) were *Pseudomonas* isolates [9]. In a study by Singh et al., in a comparison between the MicroScan and Mikrolatest Kit, 34 carbapenem-resistant Gram-negative bacteria were isolated from diverse clinical samples received by the microbiology department. These samples included blood, urine, pus, pleural fluid, sputum, bronchoalveolar lavage, and endotracheal aspirate. The most frequently encountered bacterial species were *E. coli* and *Acinetobacter* spp. [1].

Goyal et al. conducted a study to compare how colistin MICs are determined using reference BMD. MDR bacteria included A. baumannii (21.1%), P. aeruginosa (5.5%), E. coli (21.1%), and *Klebsiella* spp. (27.7%), *Citrobacter* spp. (10%), and *Enterobacter* spp. (14.4%). Among the 90 MDR bacteria tested, 23 were carbapenem-resistant *Enterobacteriaceae* (25.5%), and 10 were carbapenem-resistant *A. baumannii *(CRAB) [12]. In this study, the colistin MicroScan AST system (MIC) indicated that 91 (86.67%) cases showed an intermediate susceptibility (≤2) while 14 (13.33%) cases were classified as resistant (>4). Similarly, in the CBDE test (MIC), 100 (95.24%) cases demonstrated intermediate susceptibility (≤2), whereas five (4.76%) cases were classified as resistant (≥4).

Discrepancies in colistin susceptibility results were classified into two types of errors: MEs and VMEs. MEs occurred when the MicroScan system classified isolates as resistant, but CBDE identified them as intermediate. This could result in colistin being withheld from a patient who actually needed it, assuming MicroScan's incorrect resistance classification was followed. VMEs were observed when MicroScan classified bacterial isolates as intermediate, but CBDE revealed them as resistant. In such cases, if colistin was administered despite being genuinely resistant, based on MicroScan's incorrect intermediate result, it constituted a VME.

Regarding errors in testing, 94 out of 105 cases showed no errors. MEs were observed in 10 (9.52%) cases, while VMEs were found in only one (0.95%). In cases of MEs, out of 10, three patients received polymyxin B and one received colistin. In the VME case, the study subject did not receive colistin before sample collection but was administered meropenem and teicoplanin. The subject, a 58-year-old male with *E. coli* isolated from his urine and diagnosed with sepsis, eventually received colistin due to multiple culture reports. Singh et al. conducted a similar study comparing the MicroScan with the Mikrolatest kit for assessing in vitro colistin susceptibility. The study identified three discrepancies: two MEs were noted for *A. baumannii*, and one VME occurred for *P. aeruginosa* [1].

In the present study, results of susceptibility testing for colistin using the colistin MicroScan (MIC) and CBDE were analyzed across different bacterial species. Among the 32 cases of *A. baumannii complex*, six (18.75%) showed resistance according to the colistin MicroScan Walkaway 96 plus AST system method, with two (6.25%) displaying resistance according to CBDE, and no VMEs were observed. However, four (12.50%) cases incurred MEs, resulting in a moderate agreement (kappa value = 0.448, p = 0.002) with a categorical agreement of 87.5%. In the research by Goyal et al. comparing CBDE and BMD methods, all isolates were classified as having intermediate susceptibility to colistin according to the latest CLSI guidelines. However, five isolates (three *A. baumannii *and two *K. pneumoniae*) that had an MIC of 2 μg/mL with BMD showed an MIC of <1 μg/mL with CBDE [12].

In a study led by Lee et al., three commercial methods for assessing colistin susceptibility (Vitek 2, Etest, and MicroScan) were evaluated using 213 bloodstream isolates of *Acinetobacter* identified via gene sequencing [13]. Compared to the agar dilution reference method, both Vitek 2 and Etest demonstrated excellent categorical agreements of 99.1%. In contrast, MicroScan showed an overall agreement of 87.3%, with agreements of 95.7% for *A. baumannii* and 80.7% for non-*A. baumannii* isolates. Marlinghaus et al. assessed the accuracy of colistin susceptibility testing using the MicroScan with 327 isolates of carbapenemase-producing *Enterobacteriaceae*. Among these, 107 were resistant to colistin, and 220 were susceptible. The system failed to identify three colistin-resistant isolates, resulting in ME and VME rates of 0.9% and 2.8%, respectively [14].

Jayol et al. investigated the accuracy of three commercial BMD panels (Sensititre by ThermoFisher Diagnostics, UMIC by Biocentric, and MicroScan by Beckman Coulter) for determining colistin susceptibility in 185 Gram-negative bacilli isolates. Among these, 133 were colistin-resistant and 52 colistin-susceptible isolates, with manual BMD as the reference method. The study revealed a notable discrepancy with the MicroScan system, showing a high error rate (26.9%), mainly due to overestimated MICs for non-fermenting Gram-negative bacilli [4]. In a recent study by Pfennigwerth et al. involving 325 carbapenemase-producing *Enterobacteriaceae* species, the MicroScan WalkAway system demonstrated inadequate performance, leading to 16 MEs and 13 VMEs [15].

Screening all isolates would increase the workload with a lower yield. Therefore, we recommend routine screening for MBL production by CDT for all isolates resistant to cephalosporins and carbapenems, as this test is easy to perform and interpret. Implementing simple screening tests like CDT is a crucial step toward large-scale monitoring of these emerging resistant determinants. We present a precise, straightforward, and practical method for determining colistin MICs, which overcomes several current challenges in colistin antibiotic susceptibility testing (CBDE). As the prevalence of MDR Gram-negative bacterial infections with few treatment alternatives grows, an enhanced approach to colistin MIC determination becomes increasingly crucial. Before reporting colistin susceptibility using MicroScan, it is necessary to check the panel and compare the results. Timely servicing, validation, and upgrading of systems are important. According to our study, colistin susceptibility should be determined separately using the manual CBDE method.

Limitations

A major constraint of this study was the inability to test the *E. coli* NCTC 13846 strain, known for its mcr-1 positivity, due to its unavailability. Furthermore, the study was limited by a small sample size and did not address reproducibility. Additionally, genotypic identification for MBL was not conducted. Although PCR offers specific and accurate results, its use is limited to a few laboratories due to high costs and the global diversity of MBLs. Colistin susceptibility was not confirmed using the reference test rBMD. Furthermore, the CBDE method is labor-intensive, whereas the MicroScan MIC method is simpler to perform. Clinical correlation regarding the treatment administered to all patients based on the CBDE and MicroScan susceptibility results was not assessed in this study, and this represents an additional limitation.

## Conclusions

While the CBDE method is labor-intensive, the MicroScan MIC method is simpler to perform; however, screening all isolates using these methods would substantially increase workload with relatively low yield. Therefore, we recommend routine screening for MBL production using the CBDE for all isolates resistant to cephalosporins and carbapenems, as this test is easy to perform and interpret. Implementing such simple screening tests is a crucial step toward large-scale monitoring of emerging resistant determinants. In this study, we also present a precise, straightforward, and practical method for determining colistin MICs, addressing several current challenges in colistin susceptibility testing using CBDE. As the prevalence of MDR Gram-negative bacterial infections continues to rise, improved approaches for colistin MIC determination are increasingly important. Before reporting colistin susceptibility using the MicroScan system, it is essential to verify the panel and cross-check the results. Regular servicing, validation, and timely upgrading of automated systems are also necessary. Based on our findings, colistin susceptibility should be determined separately using the manual CBDE method.
